# Dynamic prediction methods in the BC2001 clinical trial

**DOI:** 10.1186/1745-6215-16-S2-P144

**Published:** 2015-11-16

**Authors:** Nuria Porta, M Luz Calle, Rebecca Lewis, Michelle Snape, Carey Hendron, Nicholas James, Robert Huddart, Emma Hall

**Affiliations:** 1 The Institute of Cancer Research, London, UK; 2 University of Vic, Vic, Barcelona, Spain; 3 University of Birmingham, Birmingham, UK; 4 University of Warwick, Warwick, UK; 5 University Hospitals Birmingham NHS Foundation Trust, Birmingham, UK

## 

BC2001 is the largest muscle invasive bladder cancer (MIBC) radiotherapy trial undertaken to date and showed that addition of chemotherapy to radiotherapy achieves local control of invasive disease in over 80% of patients. Secondary time to event outcomes included non-invasive loco-regional recurrence, metastasis free survival or overall survival: all modelled separately. However, patients can experience more than one event of interest; and events are likely to be related. We want to understand how the occurrence of loco-regional recurrence and cystectomy following invasive recurrence may alter the risk of metastasis or bladder cancer death.

Two approaches will be explored:

1) a multistate model for the observed evolution of patients during trial follow-up. Following randomisation (initial state), the event of interest is metastasis or death due to bladder cancer (final state). Intermediate states of non-invasive or invasive loco-regional recurrence and cystectomy, are considered. Competing non disease-related events such as non-BC deaths or second primary tumours, are included as final states. By modelling all transitions between states, the cumulative incidence of metastasis or death due to bladder cancer, given the previous event history of a patient can be derived.

2) landmark analysis of metastasis free survival. Given a fixed landmark timepoint, the status of the intermediate events by that time is assessed, and introduced as baseline covariates in the model.. Patients who have had a metastasis or died before this landmark timepoint are excluded from the analysis. Dynamic prediction can be achieved by performing landmark analysis for different landmark fixed timepoints.

